# Hospital Co-Operation in Westminster

**Published:** 1922-12

**Authors:** 


### HOSPITAL CO-OPERATION IN WESTMINSTER.

LORD CAVE'S COMMITTEE, after consideration of evidence both from hospitals which have succeeded in paying their way and from those which have failed to do so, were convinced that if the voluntary hospital was to continue to prosper it must rely not only on the large subscriptions and gifts, but also to an increasing extent on moderate and continuous contributions from all classes of the community.

In regard to any scheme of collection from wage earners the Committee thought it desirable that, in order to avoid competing appeals by different hospitals, these collections should be made by some central body on behalf of all hospitals in each area, and that the proceeds should be distributed among the hospitals on some agreed basis.

The value of such opinions given by a Committee unbiased by intimate association with the administration of hospitals, after evidence had been taken from all classes of hospitals in England, Scotland and Wales, is, of course, obvious, and very careful attention was given to such opinions by a Sub-committee appointed for the purpose by the Governors of Westminster Hospital.

Consequently, in July, 1921, by direction of the Sub-committee, a letter was addressed to all voluntary hospitals in Westminster, inviting co-operation in appeals to Westminster people of all classes to support their local hospitals.

The ideal form of co-operation aimed at included, it is understood :—

(i) *Finance*—under which head come the following :— Weekly collections from wage-earning classes. Appeals for annual subscriptions, donations, box collections ; special appeals (either for ordinary or special purposes, such as building) ; bazaars, entertainments, Flag Days, &c.
(ii) *Buying Facilities* : The advantages of buying in larger quantities. Also the exchange of views, &c., as to working costs.
(iii) *Working agreement*, whereby a hospital would get the use of any empty beds there might be in another hospital for emergency cases, &c., and it was considered that there were three degrees in which co-operation might be carried out, viz. :—

(*a*) A complete co-operation between all the hospitals within the area, under which, while still maintaining their separate identity, they would, for all appropriate purposes, be amalgamated, in the sense of working together.
(*b*) A limited co-operation between one or more hospitals for joint action in connection with the items set forth in (i), (ii), and (iii) above, and
(*c*) A general working arrangement limited to specific items.

In deference to the wishes of King Edward's Hospital Fund, and in order not to interfere with the efforts of the Combined Hospitals Appeal, the consideration of the proposals was not proceeded with, but at meetings of representatives of the majority of the hospitals of Westminster the following arrangements, it is understood, have been made :—

(i) Clearly defined areas in which the individual hospitals shall work have been agreed upon. This division of Westminster, although necessary administratively, has not interfered in any way with the co-ordination of the work of the hospitals ;
(ii) A basis of distribution of moneys collected has been agreed upon. The basis is determined chiefly by the number of occupied beds and attendances of out-patients, but exception is made in the case of hospitals without beds where an increased allowance is made per attendance of out-patients ;
(iii) A Committee representative of tradesmen, bankers, business men, labour, religious bodies, the British Red Cross Society, League of Mercy, the theatrical world, and the Westminster Council, has been formed under the chairmanship of the Mayor of Westminster.

### The First Results Gained.

Co-operation took definite form in connection with the Hospitals of London Combined Appeal. It was at once realised that this group of hospitals, which comprised two general hospitals and eight special hospitals specially adapted for the treatment of almost every form of disease, could together make a very strong claim through the Mayor of Westminster's Committee to the citizens of Westminster ; and support from all classes was obtained which would not have been given to any one particular hospital. The position of Westminster in the Boroughs and Cities' lists of donations, and the great success of the Westminster collections on the Flag Days support the above statement.

### The Prospects of Local Support.

The Mayor and his Committee desire still further co-operation with their hospitals, and the hospitals have therefore now been asked if they will invite the Mayor or one member of his Committee to serve on the Executive Committee of the hospital.

The scheme of weekly collections from the wage-earning classes, which it was originally intended to put into operation in Westminster, was really no more than a systematising of the box collections, and no *quid pro quo* was offered. Providing the principles of the Hospitals Savings Association scheme are acceptable to the Committees of the various hospitals in Westminster, it is hoped that the local organisation and administration of the scheme will be undertaken by or through the Mayor of Westminster's Committee, thereby maintaining the idea of Westminster hospitals supported by their own people.

### Typical Difficulties.

The chief local difficulties have been (i) the reluctance of the many Government offices in the area to support any charities but national ones ; (ii) the fact that a very large number of wage earners in Westminster contribute a very small sum per week to the Hospital Saturday Fund. There are few difficulties, however, that cannot be overcome when the majority of the hospitals of a City supported by their Mayor present a united appeal, and much progress has already been made in overcoming the first difficulty.

The second difficulty is far more serious, as the hospitals do not wish to interfere in any way with the work which has been carried on in the area for some time by the Hospital Saturday Fund. It is hoped, however, that when distributing their funds the Hospital Saturday Fund will consider a proposal that some part of the grant shall be distributed on the basis of the number of contributors in the area served by the hospital.

The following is evidence that local patriotism is worth cultivating, even in London: At Westminster Hospital, where for some time endeavours have been made to bring home to the people the value of the work done for the sick poor and the duty of local people to support their hospital, the workmen's and office collections have increased three-fold in the past eighteen months; over £600 (new money) has been handed to the hospital from entertainments organised by local tradesmen and social clubs; local supporters contributed nearly £500 in money and over 2,000 lb. weight in food at a Pound Day recently held, and the income from regular annual subscriptions from firms and residents in the neighbourhood is increasing.

Apart from the defined objects of co-operation, the meetings have enabled representatives of the various hospitals to get to know one another and some, perhaps all, have admittedly benefited from discussions in common and joint work.

Owing to the amount of space which, in accordance with our traditions, we have devoted in this number to the special needs of our Hospitals, it has been necessary to interrupt the series of interviews with the Public Health Authorities which have been appearing regularly for some months. They will be resumed in our next and subsequent issues, which will also contain other special Public Health features.

